# Targeted spectroscopy in the eye fundus

**DOI:** 10.1117/1.JBO.28.12.126004

**Published:** 2023-12-15

**Authors:** Nicolas Lapointe, Cléophace Akitegetse, Jasmine Poirier, Maxime Picard, Patrick Sauvageau, Dominic Sauvageau

**Affiliations:** a Zilia Inc., Quebec City, Québec, Canada; b University of Alberta, Department of Chemical and Materials Engineering, Edmonton, Alberta, Canada

**Keywords:** ocular spectroscopy, diffuse reflectance, fluorescence, biomarkers, oximetry

## Abstract

**Significance:**

The assessment of biomarkers in the eye is rapidly gaining traction for the screening, diagnosis, and monitoring of ocular and neurological diseases. Targeted ocular spectroscopy is a technology that enables concurrent imaging of the eye fundus and analysis of high-quality spectra from a targeted region within the imaged area. This provides structural, compositional, and functional information of specific regions of the eye fundus from a non-invasive approach to ocular biomarker detection.

**Aim:**

The aim of our study was to demonstrate the multimodal functionality and validation of targeted ocular spectroscopy. This was done *in vitro*, using a reference target and a model eye, and *in vivo*.

**Approach:**

Images and spectra from different regions of a reference target and a model eye were acquired and analyzed to validate the system. Targeted ocular fluorescence spectroscopy was also demonstrated with the same model. Subsequently, *in vivo* imaging and diffuse reflectance spectra were acquired to assess blood oxygen saturation in the optic nerve head and the parafovea of healthy subjects.

**Results:**

Tests conducted with the reference target showed accurate spectral analysis within specific areas of the imaging space. In the model eye, distinct spectral signatures were observed for the optic disc, blood vessels, the retina, and the macula, consistent with the variations in tissue composition and functions between these regions. An ocular oximetry algorithm was applied to *in vivo* spectra from the optic nerve head and parafovea of healthy patients, showing significant differences in blood oxygen saturation. Finally, targeted fluorescence spectral analysis was performed *in vitro*.

**Conclusions:**

Diffuse reflectance and fluorescence spectroscopy in specific regions of the eye fundus open the door to a whole new range of monitoring and diagnostic capabilities, from assessment of oxygenation in glaucoma and diabetic retinopathy to photo-oxidation and photodegradation in age-related macular degeneration.

## 1 Introduction

Ocular diseases generally involve specific structural and functional changes in the eye fundus. For example, a progressive degeneration of ganglion cells characterizes glaucoma,^1^ accumulation of drusen and autofluorescent pigments are observed in age-related macular degeneration (AMD),^2^ and microaneurysms, retinal hemorrhages, and vascular abnormalities are associated with diabetic retinopathy (DR).^3^ Morphological and functional changes in the eye fundus are, however, not strictly restricted to vision-related diseases. Recent studies have shown that certain neurological diseases, such as Parkinson’s and Alzheimer’s, lead to observable changes in the retina, such as thinning of the retinal nerve fiber layer (RNFL) and changes in hemodynamics.^4^^–^^10^

Spectral analysis of light reflected or emitted by the retinal tissue may provide information on changes in the retina that cannot be uncovered through typical fundus color imaging or optical coherence tomography (OCT). Thus, this approach has the potential to act as a complement to current assessment tools for a number of research and clinical applications. For instance, the accumulation of lipofuscin in the retinal pigment epithelium (RPE) causes fundus autofluorescence;^11^^,^^12^ structural changes in RNFL impact retinal reflectance;^13^^,^^14^ the absorption spectrum of blood varies according to the ratio of its content in oxygenated and reduced hemoglobin;^15^ and melanin has a characteristic spectral profile in the visible spectral range.^1^ The resulting localized spectral changes have different outcomes that impact the optical properties of retinal tissues, which can be assessed through diffuse reflectance and fluorescence spectroscopy.

Over the years, multiple groups have worked on developing ocular diffuse reflectance spectroscopy methods.^16^^–^^19^ For example, Van Norren and Tiemeijer^16^ used a retinal densitometer to measure the spectral reflectance of the optic disc, the peripheral retina, and the fovea with an angular resolution of 2.5 deg. The authors were able to establish that reflectance was higher in the red range than in the blue range and developed a model to explain their measurements. Delori and Pflibsen used a modified fundus camera to acquire retinal reflectance spectra from 450 to 800 nm. They achieved an angular resolution varying from 1 deg to 4 deg by placing an aperture in the illumination path to limit the illuminated area.^17^ A grating monochromator and a camera were used for spectral acquisition, and a fixation target was used to change the measurement site. Later, Delori^17^ developed a system allowing for the acquisition of both the intrinsic fluorescence spectrum and the reflectance of the eye fundus in the spectral range of 500 to 800 nm using a modified ocular fluorometer. To achieve this, an aperture in the image plane of the retina was used to define the sampling area. Hammer^18^ used a slit-shaped stop in an image plane of the eye fundus to acquire reflectance spectra of horizontal areas. Diaconu^19^ and Vucea et al.^20^ proposed a system using a holed-mirror located in an image plane to reflect light coming from the fundus to a camera with the same intent. The light that passed through the small hole was acquired by a spectrometer, and thus the spectral sampling area was the center of the image, where a black circular region was visible due to the hole in the image plane. Although allowing for the acquisition of a localized spectral signal in the eye fundus, the aforementioned techniques lack flexibility in the sense that the acquisition area is only adjusted by the fixation of the subject, which can be laborious when fine positioning is required. Only the method developed by Diaconu^20^ allowed for simultaneous spectral acquisition and visualization of the acquisition area in the eye fundus.

Other technologies, such as hyperspectral imaging and visible-light coherence tomography (vis-OCT), have been recently used to monitor similar variations in optical properties in the eye fundus. Hyperspectral imaging allows for the acquisition of eye fundus images with several color channels, such that each pixel contains spectral information. Some of these systems consist of retinal cameras in which the transmitted wavelength band is controlled by a tunable filter applied either in imaging or illumination to construct the hyperspectral cube.^21^^,^^22^ In other cases, a diffraction grating was used to separate colors, each of which collected by a dedicated area of the detector.^23^^,^^24^ All of these systems face a trade-off between spectral resolution, acquisition speed, and spatial resolution; image registration is generally also required. In vis-OCT,^25^ a supercontinuum light source in the visible spectral range enables higher resolution and acquisitions with absorption contrast information. In this case, there is an additional trade-off between the illumination intensity and the acquisition time to obtain enough signal for analysis.

Considering the growing interest and the heterogeneity in the structural, functional, and compositional properties of the eye fundus, a spectral assessment of specific regions of the eye fundus is relevant. We describe, here, a system that allows for the visualization of the eye fundus while simultaneously acquiring full visible diffuse reflectance or fluorescence spectra from a targeted location of the eye fundus. The user can select a target and move it to any location within the eye fundus region being imaged without any realignment or change of the fixation target while continuously receiving spectral information of the targeted sampled area. Such assessments can potentially provide important information for the detection, diagnosis, and/or monitoring of ocular and other diseases. Demonstrations of diffuse reflectance in the visible range (*in vitro* and *in vivo*) and fluorescence (*in vitro*) were performed. *In vivo* ocular oximetry assessments were also performed in healthy patients using previously established algorithms.^26^

## 2 Materials and Methods

### 2.1 Targeted Spectroscopy

**Fig. 1:**
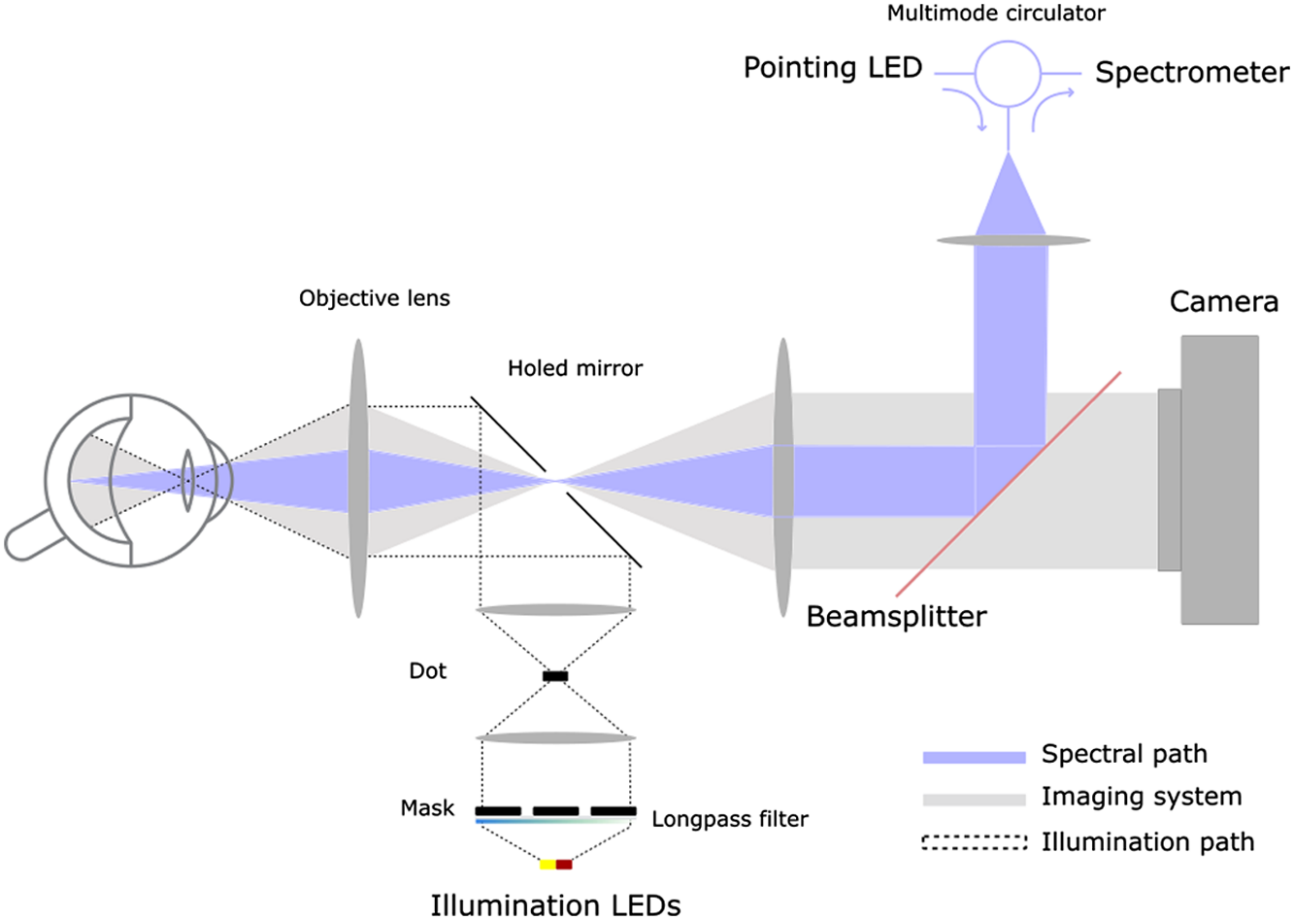
Targeted spectroscopy optical layout. Illumination LEDs illuminate the retina. Reflected light is directed partially toward the camera and partially toward the spectrometer. The pointing LED is projected onto the eye fundus to identify the ROSA. Light to the spectrometer comes from the ROSA as it shares the same optical path as the pointing LED.

The optical layout used for concurrent imaging and spectroscopy is based on the Zilia Ocular platform (Fig. 1). Light from two 5000K white light emitting diodes (LEDs) (YJ-BC-3030-G04, Yujileds, Beijing, China) and two NIR LEDs centered at 785 nm (SST-10-FR-B90-H730, Luminus, Sunnyvale, United States) is magnified through a custom-made 4-f system and reflected by a holed-mirror through the pupil using an objective lens, as can be seen in the illumination path of Fig. 1. The white light was filtered via a long-pass 495-nm filter (AT495lp, Chroma). The filter was added to the illumination channel to reduce blue light exposition for the patient, allowing for greater light intensity in the 500 to 750 nm range without impacting eye safety or oxygen saturation measurements. The user can choose to use the white or NIR LEDs for fundus illumination. The output power of both light sources was adjusted to optimize the signal intensity, up to 3.38 mW for the white source and up to 1.98 mW for the NIR source. This corresponded to a maximum irradiance on the retina of 4.51 and $2.64\;\mathrm{mW}/cm^{2}$, respectively, and to a maximum irradiance on the cornea of 16.36 and $9.58\;\mathrm{mW}/cm^{2}$, respectively. The system was developed according to ANSI Z80.36-2021 for eye safety purposes. To reduce reflections from the cornea, a mask was placed in a plane conjugate to the pupil, and reflections on the objective lens were minimized by the use of a black dot in a plane conjugate to the back surface of the objective lens. The light reflected on the eye fundus was collected by the objective lens, passed through the hole of the holed-mirror, and projected to the camera via another 4-f system. A sputter-coated, nonpolarizing beam splitter (CHROMA, PN 21020, 80/20) was placed at the Fourier plane of the imaging system to project a fraction of the light oriented toward the camera in the direction of a multimode circulator (Castor Optics Inc., WMC2L1-C). This fraction of light was then sent through a group of lenses and was collected and analyzed by a spectrometer with a resolution of 2.3 nm and a spectral response range from 340 to 830 nm.

To identify the region of spectral acquisition (ROSA)—an area of 1.5 deg on the eye fundus for which spectral analysis was performed—a 730-nm LED, referred to as the pointing LED (SST-10-FR-B90-H730, Luminus, Sunnyvale, United States) was connected to the remaining port of the multimode circulator. This configuration allowed the light collected by the spectrometer and that emitted by the pointing LED to share the same optical path, from the exit of the circulator to the eye fundus. Thus, when the pointing LED was activated, it illuminated the exact position of the ROSA, and the camera could capture an image of its location. The non-polarizing beam splitter was mounted in such a way that it could rotate about its axis using two linear actuators. This movement enabled displacement of the ROSA within the imaged field of view.

**Fig. 2:**
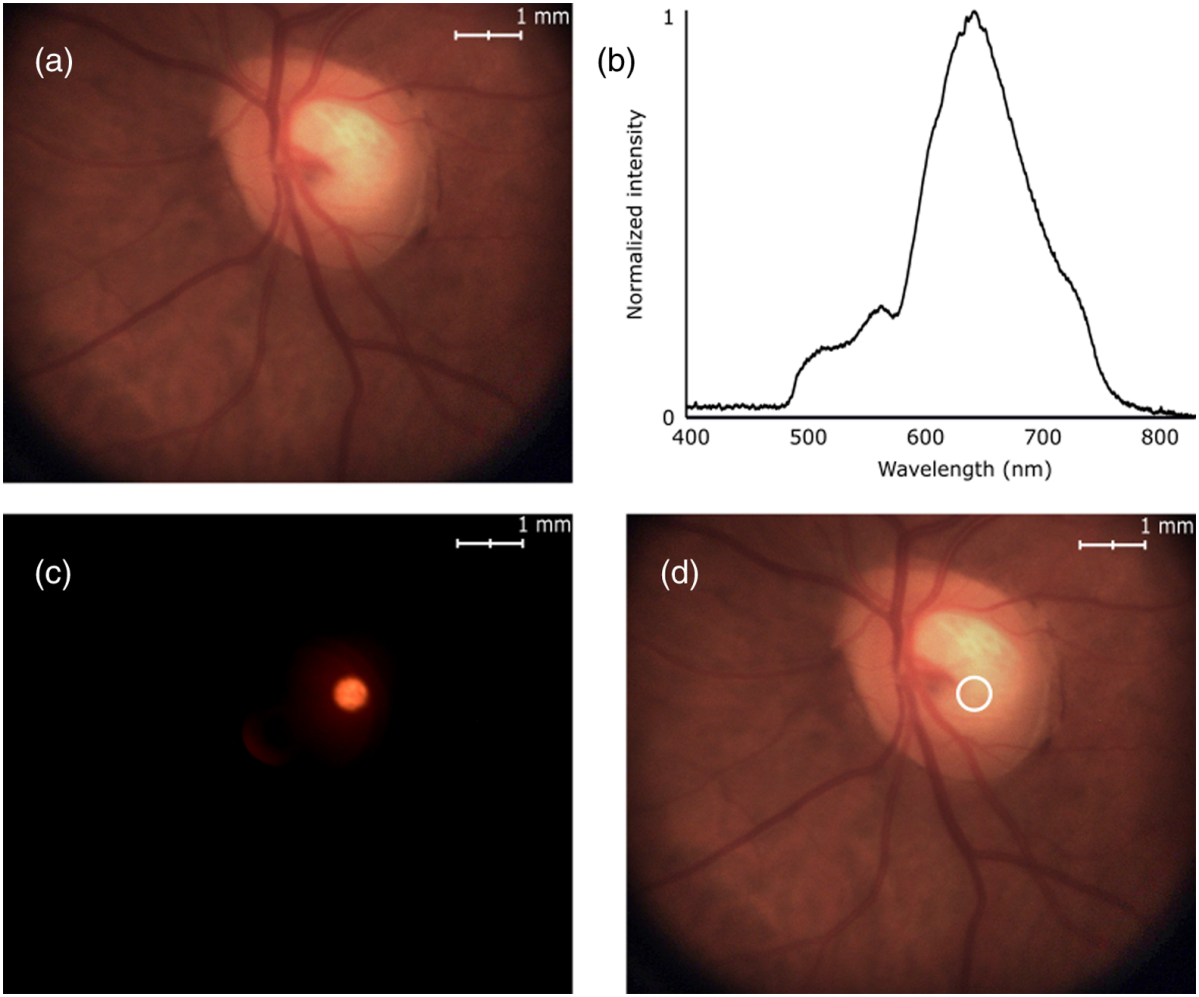
Principles of operation of targeted spectroscopy, enabling continuous, real-time imaging and spectral acquisitions. (a) A white LED is used to illuminate the eye fundus, and an image is acquired by the camera. (b) A spectrum is simultaneously acquired by the spectrometer. (c) The white LED is turned off, and the camera acquires an image of the pointing LED projected onto the eye fundus, thus defining the ROSA. (d) An image of the eye fundus overlaid with the location of the ROSA is shown to the user.

A two-step acquisition sequence was developed to combine imaging and targeted spectroscopy. First, the illumination LED was turned on, illuminating the eye fundus and enabling simultaneous acquisition of an image by the camera and of a diffuse reflectance spectrum (DRS) by the spectrometer [Figs. 2(a) and 2(b)]. Then the illumination LED was turned off, and an image was acquired with only the pointing LED turned on [Fig. 2(c)]. This enabled the identification and segmentation of the ROSA, while preventing crosstalk in the optical fiber. The segmentation of the ROSA image was used to identify the location of the DRS acquisition area [Fig. 2(d)]. At least one image of the eye fundus was recorded for each spectrum acquired. If the image was blurry or if the position of the ROSA was displaced beyond half a radius of its intended target location—due to saccades, closing of the pupil, or other eye movements^27^—the corresponding spectrum was discarded, and the rest of the acquisition proceeded.

### 2.2 Reference Target

To demonstrate the ability of the system to acquire a spectrum from a selected region, an ultrahigh-definition screen (HP V28 4K, 3840  pixels×2160  pixels and pixel pitch of 0.116 mm) was used to display a reference target. The target displayed on the screen consisted of an 8×8 grid with 8 different colors [Fig. 4(a)]. Each chosen color consisted of an RGB color code, with each component at either 0 or maximum intensity. The fundus camera with a 150-mm lens (Edmund Optics, PN #47-352) at its entrance pupil was positioned in front of the screen (Fig. S1 in the Supplementary Material). For these experiments, the illumination of the fundus camera was switched off, and only the light emitted by the screen was collected for spectral analysis.

### 2.3 Model Eye

**Fig. 3:**
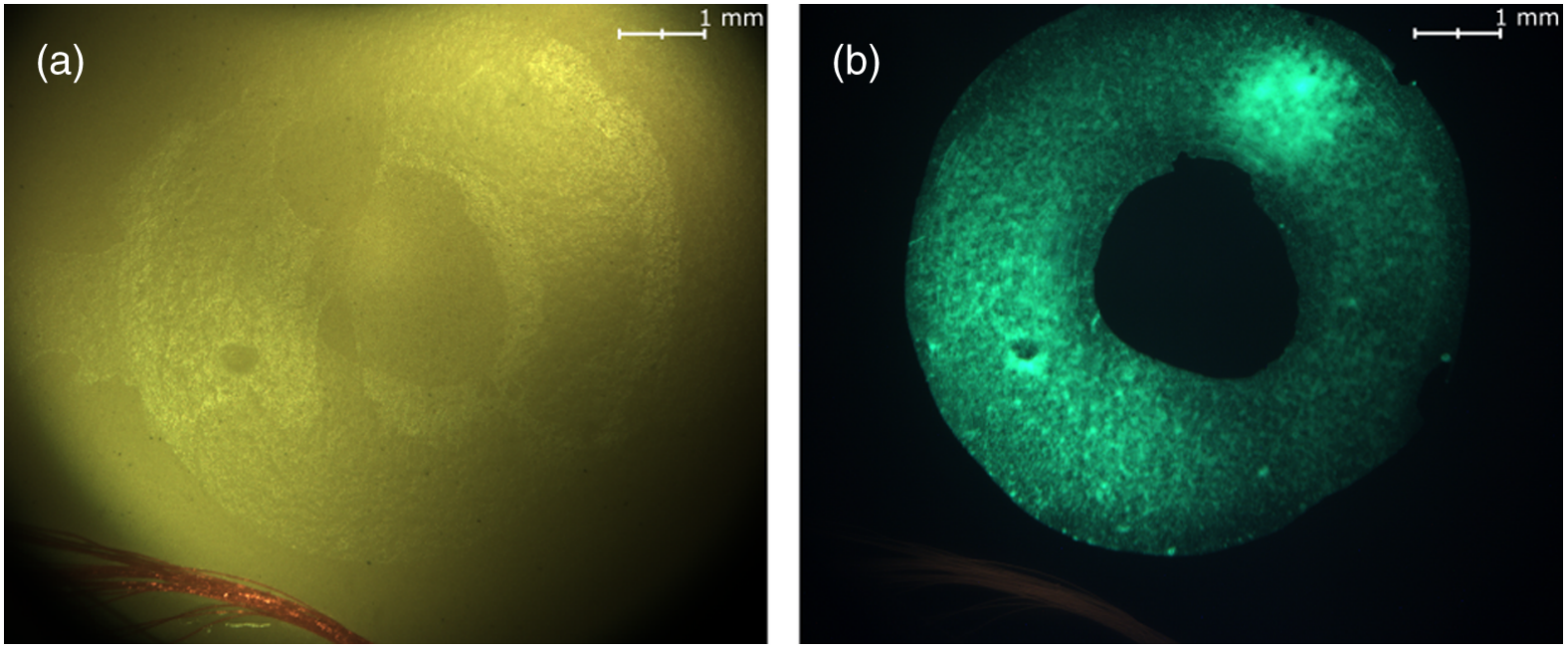
Ocular imaging eye model OEMI-7. (a) White reflectance image, in which a simulated macula and blood vessels (in bottom left) can be seen. (b) Fluorescence imaging, in which the circular fluorescent pattern can be seen.

An imaging Eye Model (OEMI-7, Optical Instruments, Bellevue, Washington, United States) with a 7-mm pupil was used to validate diffuse reflectance spectroscopy acquisitions (Fig. 3). The OEMI-7 eye model is designed to accurately simulate the human eye; it includes an anterior chamber and a crystalline lens. It displays a representation of the macula, a foreign body, the optic disc, and blood vessels [Fig. 3(a)]. Moreover, the model has fluorescent patterns, allowing for simulated fluorescence imaging [Fig. 3(b)].

### 2.4 Fluorescence Acquisition

Slight modifications of the optical design enabled the assessment of fluorescence through imaging and spectroscopy. A bandpass filter (AT460/50, Chroma Technology, Bellows Falls, Vermont, United States) was inserted in the illumination path to isolate the excitation illumination for green fluorescence imaging. A longpass filter (AT495LP, Chroma Technology, Bellows Falls, Vermont, United States) was also added to the system, behind the holed mirror. This filter enabled exclusive imaging and spectral acquisition of light emitted by fluorescence.

### 2.5 Spectral Analysis

Each acquired raw spectrum went through the same three processing steps. (1) The spectral contribution of ambient light—previously defined in a calibration step in which acquisitions were made without any illumination from the system—were removed from the spectrum. (2) The effect of the illumination source spectrum—previously defined in a control step for which the spectrum of a flat white sample (Labsphere, Spectralon) was acquired—was accounted for. (3) The spectrum was normalized by the standard deviation of the raw signal from 420 to 700 nm (effective range of the white color spectrum) to correct for differences in signal intensity. The background was computed by averaging the spectral intensity between 350 and 400 nm, where the only spectral contribution was electrical noise from the spectrometer because no UV light was present in the signal. Since the absolute absorbance values were not directly compared, a scaling factor enhanced the spectral differences.

### 2.6 Human Subjects

Eight non-smoking, healthy adults (mean age: 32 years old, range: 27 to 35 years, 4 males/4 females) were enrolled. They presented no systemic disease and took no medication. For all subjects, ophthalmic examination—including slit lamp biomicroscopy, measurement of visual acuity, objective refraction, Goldmann tonometry, and funduscopic examination—produced normal findings. Exclusion criteria were ocular disease, acute infection, known diabetes mellitus, epilepsy, history of systemic hypertension, or abnormal clinical optic disc appearance. After the examination, the right eye was dilated using tropicamide 1% and phenylephrine 2.5%. A 20-min acclimation period followed. The protocol of the study followed the guidelines of the Declaration of Helsinki. Signed informed consent was obtained from all subjects after the nature of the study was explained, and all participants were informed of their right to withdraw from experimentation at any point in the study.

### 2.7 Calculation of Oximetry

An ocular oximetry algorithm, previously developed for the determination of blood oxygen saturation in the tissues of the eye fundus (${\mathrm{StO}}_{2}$),^26^ was used to process diffuse reflectance spectra acquired *in vivo*. This algorithm is based on the modified Beer–Lambert law and includes contributions from erythrocyte light scattering,^28^ a scaling term, retinal melanin, the crystalline lens, oxyhemoglobin, and deoxyhemoglobin. Equation 1 was used for the calculation of oxy- and deoxyhemoglobin:

$$\mathrm{OD}(\lambda )=c_{o}+c_{1}\;\log\;\frac{1}{\lambda }+c_{\mathrm{HbO}}\varepsilon_{\mathrm{HbO}}(\lambda )+c_{\mathrm{HbR}}\varepsilon_{\mathrm{HbR}}(\lambda )+c_{\mathrm{Mel}}\varepsilon_{\mathrm{Mel}}(\lambda )+c_{S}S_{\text{lens}}(\lambda ),$$ (1)

where OD(λ) is the optical density at a given wavelength; $c_{\mathrm{HbO}}$, $c_{\mathrm{HbR}}$, and, $c_{\mathrm{Mel}}$ are the molar concentrations of oxyhemoglobin, deoxyhemoglobin, and melanin, respectively; $\varepsilon_{\mathrm{HbO}}(\lambda )$, $\varepsilon_{\mathrm{HbR}}(\lambda )$ and, $\varepsilon_{\mathrm{Mel}}(\lambda )$ are the molar extinction coefficients at the given wavelength for oxyhemoglobin, deoxyhemoglobin, and melanin, respectively; and $S_{\text{lens}}(\lambda )$ is the crystalline lens reference optical density at the given wavelength. Hemoglobin molar extinction coefficients were obtained from Ref. 29, melanin coefficients were from Ref. 30, and the optical density spectrum of the crystalline lens was from Ref. 31.

Blood oxygen saturation (${\mathrm{StO}}_{2}$) was calculated as the ratio of oxygenated hemoglobin to total hemoglobin:

$${\mathrm{StO}}_{2}=\frac{c_{\mathrm{HbO}}}{c_{\mathrm{HbO}}+c_{\mathrm{HbR}}}\times 100\%.$$ (2)

## 3 Results

### 3.1 Spectral Acquisition of a Targeted Region

**Fig. 4:**
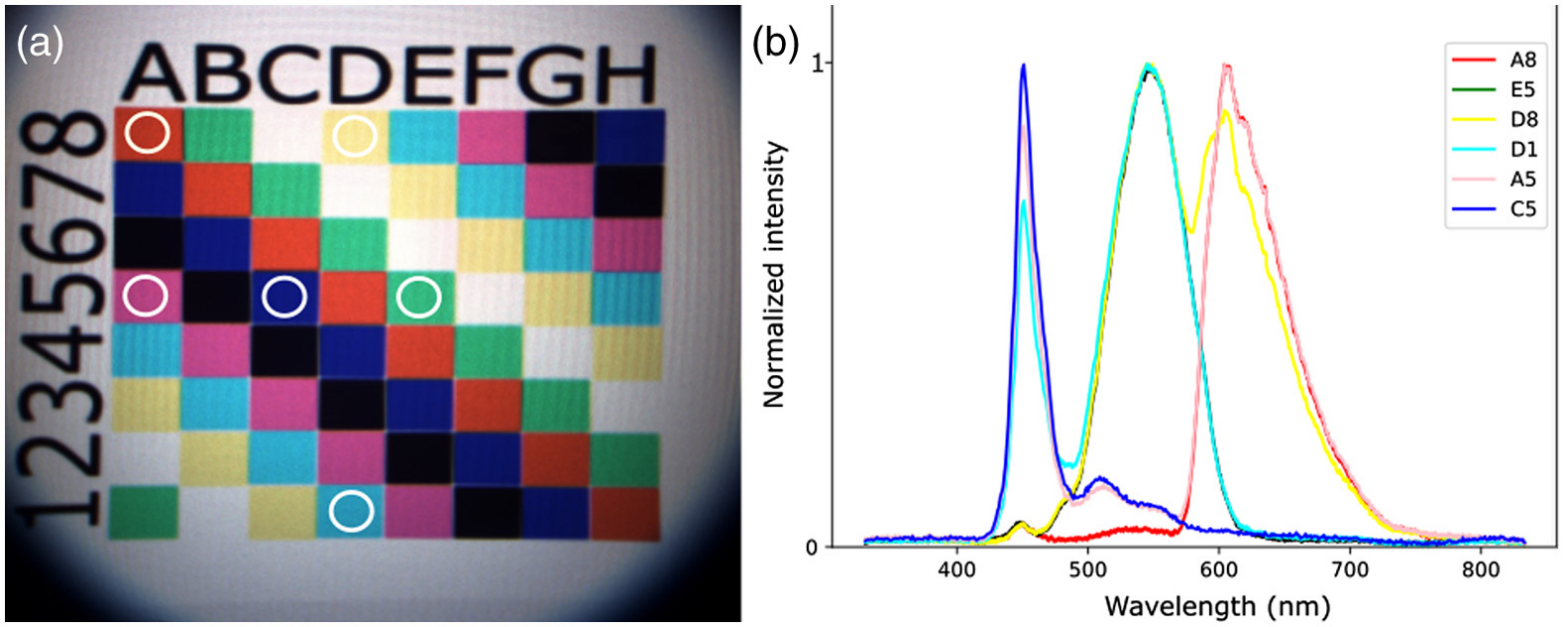
Reference target for targeted spectroscopy. (a) The white circle overlays represent the different projections of the pointing LED for the regions tested. (b) The spectra, normalized to 1, associated with each region tested, corresponding to different colors. The screen used for the reference target was the only illumination source (i.e., the illumination sources from the system were turned off).

The ability of the system to acquire a spectrum from a specific targeted region within the imaging field of view was tested using the reference target described in Sec. 2.2. Spectra were acquired by moving the ROSA to six different locations within the field of view of the reference target [white circles in Fig. 4(a)]. The spectral profiles of the different regions tested, each representing a different color, are shown in Fig. 4(b). Spectra from eight independent acquisitions taken at location D1 were overlaid (Fig. S2 in the Supplementary Material) as an example of the reproducibility of the spectral acquisitions.

**Fig. 5:**
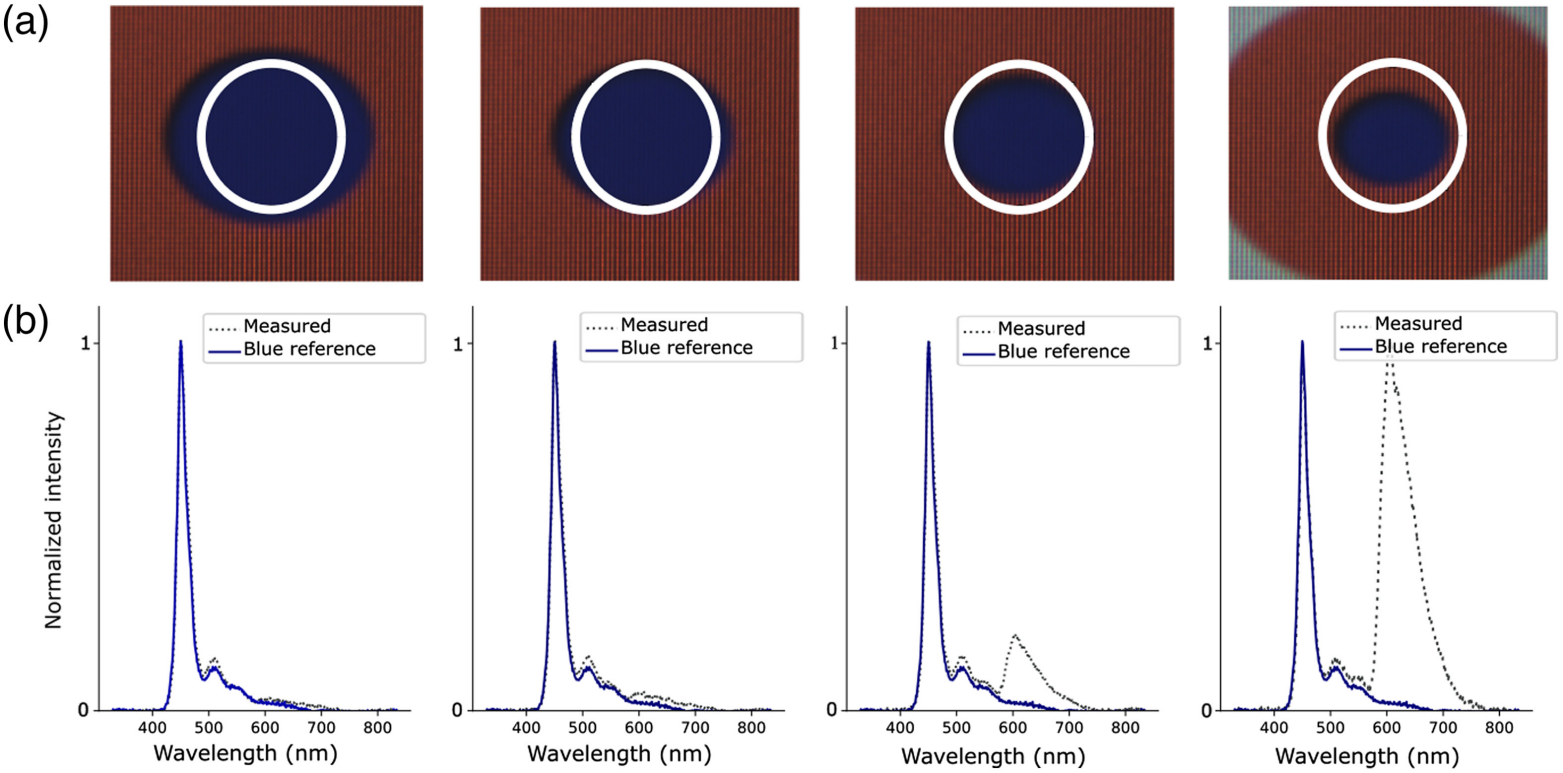
Testing of the overlay of the illumination LED and ROSA for targeted spectroscopy using reference targets. (a) Visualization of the ROSA overlays. From left to right, the concentricity of the ROSA, depicted with the white circle overlay, was decreased. (b) Spectroscopy results, normalized to 1, compared with a blue reference spectrum. Results show a decreasing contribution of the blue circle and an increasing contribution from the surrounding red area. The screen used for the reference target was the only illumination source (i.e., the illumination sources from the system were turned off).

To confirm that the region illuminated by the pointing LED precisely matched the ROSA, spectra were acquired on two concentric circles of different colors (red and blue) and dimensions (Fig. 5). The inner circle diameter was set to different values to represent limited cases of the ROSA diameter. The ROSA was centered on the internal circle. The contribution of the red peak, at 625 nm, was only detected in the third and fourth acquisitions, where the ROSA slightly overlapped with the red color, indicating that the measure was localized within the area defined by the pointing light.

### 3.2 Reflectance Acquisition in Model Eye

**Fig. 6:**
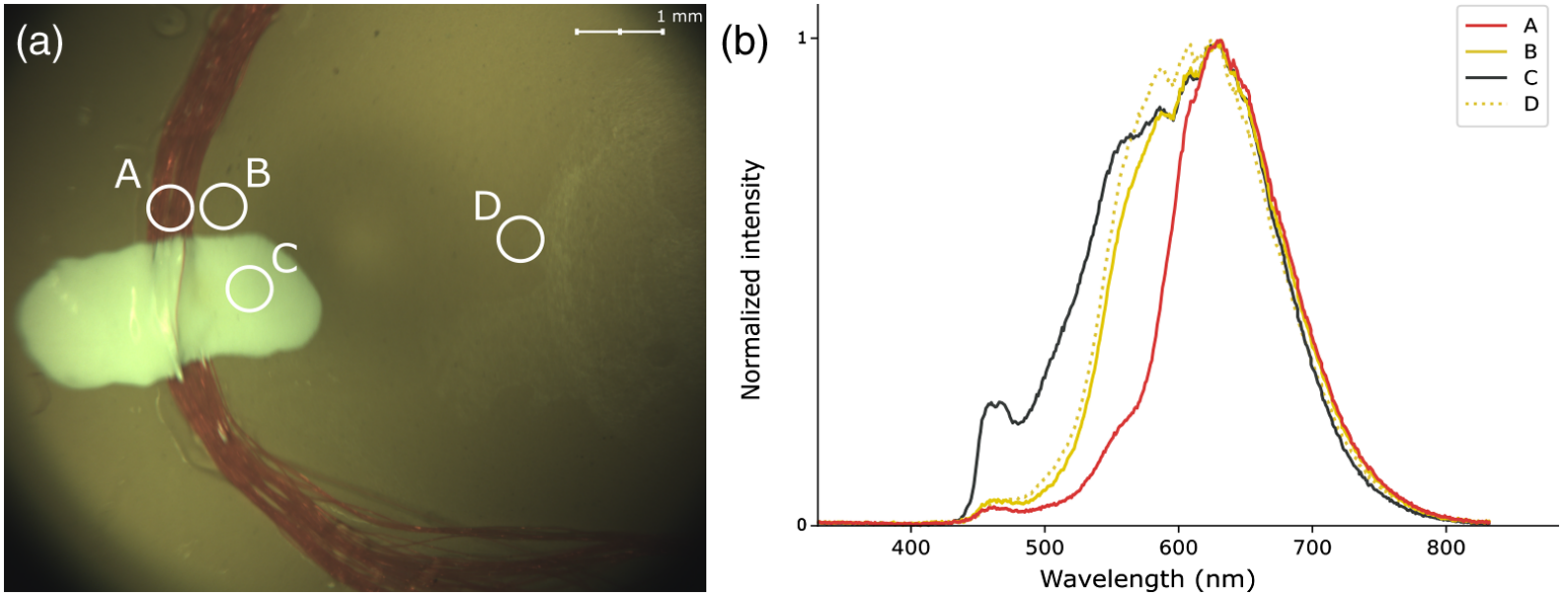
OEMI-7 model eye for targeted spectroscopy. (a) Imaging of the model and the four regions of diffuse reflectance spectral acquisitions (A, blood vessels; B, retina near the optic nerve head; C, optic nerve head; and D, retina far from the optic nerve head). (b) Reflectance spectra, normalized to 1, associated with each region of acquisition.

The model eye was used to demonstrate the capacity of the system to acquire reflectance spectra from various regions using the illumination source of the system. Four different regions were targeted—corresponding to blood vessels (A), retina near the optic nerve head (B), optic nerve head (C), and retina far from the optic nerve head (D)—and their spectra were compared (Fig. 6). Regions B and D, which both represent the retina, showed similar reflectance spectra, with only small variations detected. However, clear differences were observed between region A and region C, corresponding to the blood vessels and the optic nerve head, respectively.

### 3.3 Fluorescence Acquisition in Model Eye

**Fig. 7:**
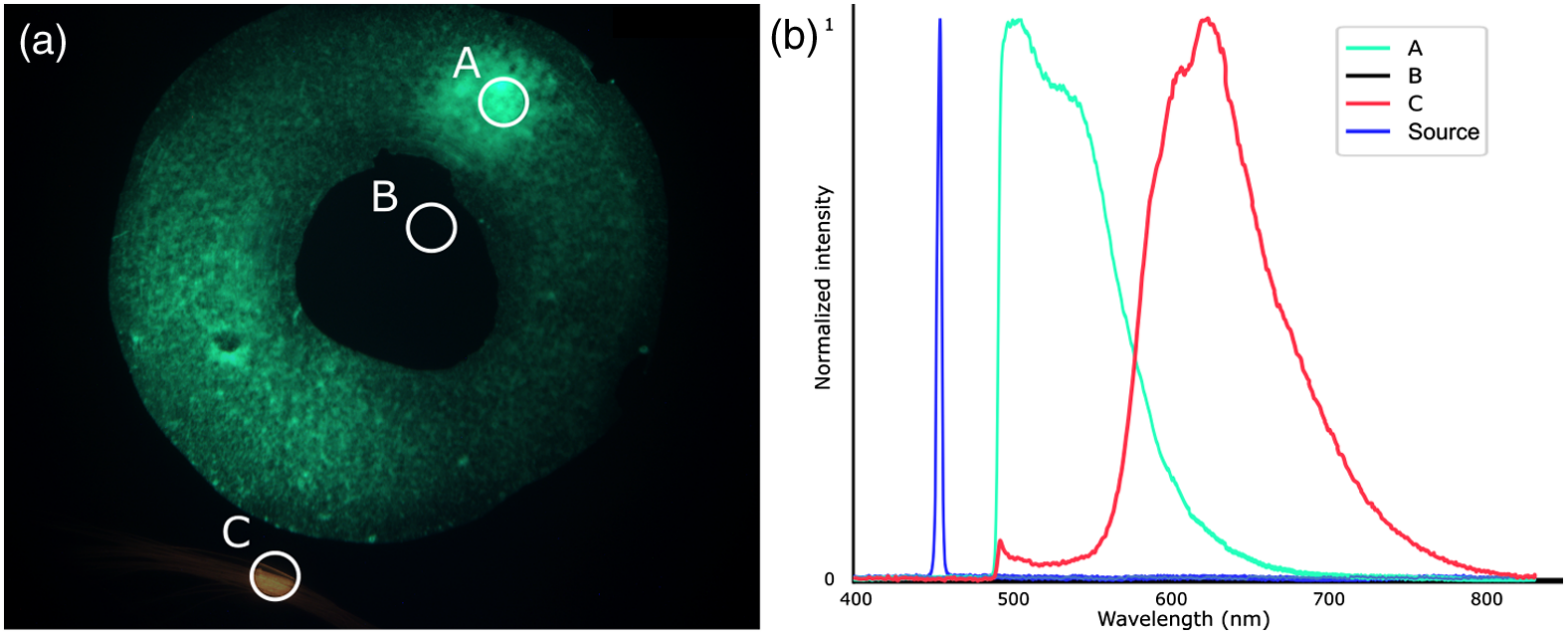
OEMI-7 model eye for fluorescence spectroscopy. (a) Fluorescence imaging of the model and identification of the three regions targeted for fluorescence spectral acquisitions. (b) Spectra associated with each targeted region (A, green fluorescence; B, no fluorescence; and C, red fluorescence) and with the excitation illumination source. Spectra were normalized to 1, except for the spectrum of region B for which no signal was detected.

The model eye used to validate that diffuse reflectance acquisitions can also be used to perform targeted fluorescence analysis. Different regions were targeted and analyzed for fluorescence (Fig. 7). Region A corresponded to a region of high-intensity green fluorescence located in the model macula. Region B corresponded to a region located in the fovea, where no fluorescence signal was visible from the wide field image. Region C corresponded to model blood vessels, with some red fluorescence, located on the simulated retina. Figure 7(b) shows clear spectral differences between the emitted spectral profiles of regions A and C. The fluorescence signal was only emitted from the targeted region if the targeted region possessed fluorescence capacities, which was observed in regions A and C.

### 3.4 Reflectance of the Human Eye Fundus

**Fig. 8:**
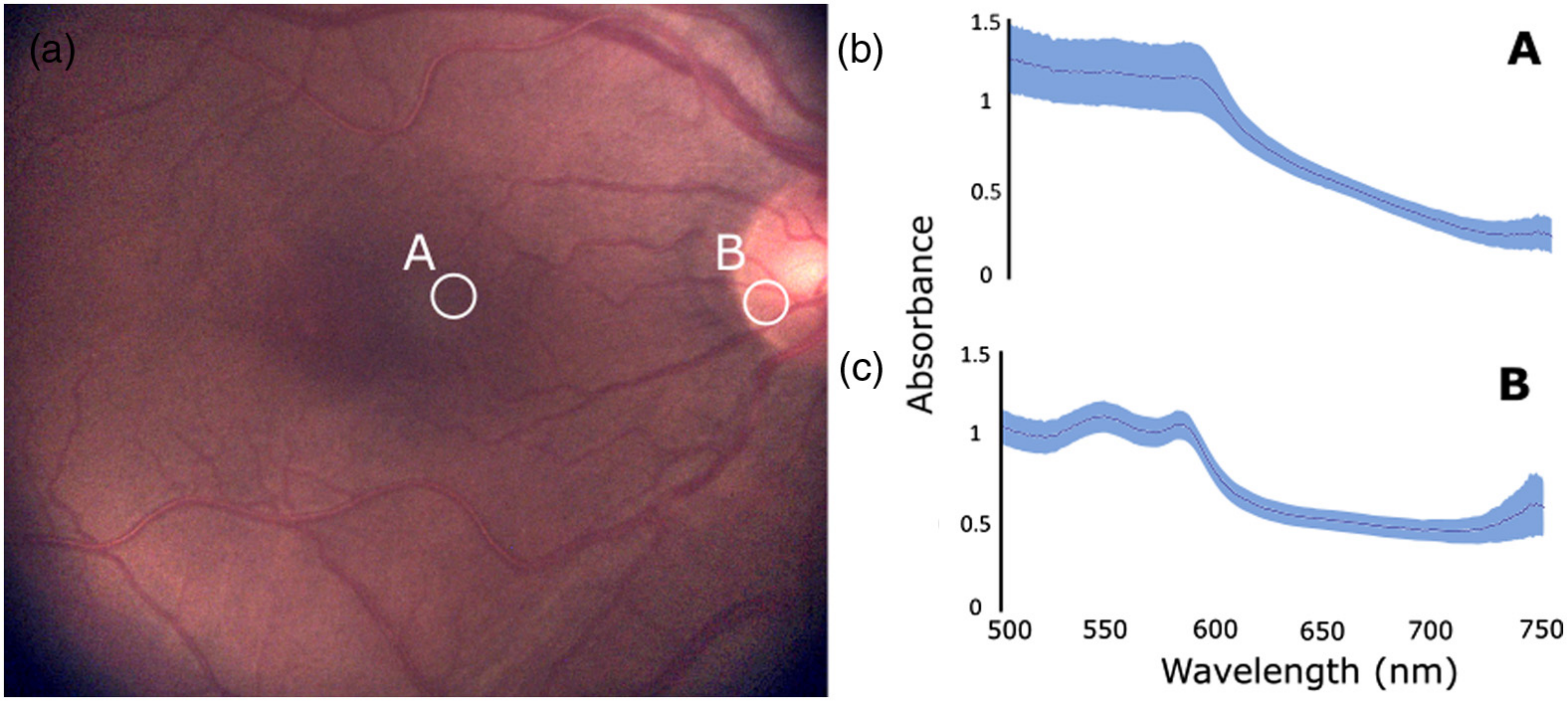
Diffuse reflectance spectral acquisitions on healthy human subjects. (a) Locations of the spectral acquisitions performed in healthy subjects: A, parafovea; B, optic nerve head. (b) Average absorbance spectra for location A over the eight subjects accounting for interindividual variability. (c) Average absorbance spectra for location B over the eight subjects accounting for interindividual variability.

Spectral acquisition was performed in two different regions of the eye fundus—the parafovea and optic nerve head, each anatomically different and having different optical properties—of eight healthy subjects. Figure 8 shows the locations of the acquisitions and the average spectral signatures obtained for the eight subjects. The blue band represents the standard deviation of the spectra between the different subjects to show interindividual variability. The reproducibility of spectra for a given acquisition is shown in Fig. S3 in the Supplementary Material.

Light intensity was adjusted for every acquisition (subject and location) to cover the whole dynamic range of the spectrometer for a given integration time. Five-second acquisitions, corresponding to 13 acquired spectra, were made at each location. The combined integration time of the spectrometer and the camera was 370 ms (250 ms for the spectrometer and 120 ms for the camera), resulting in a frequency of acquisition of 2.70 Hz. All acquisitions, taken from the same location, were averaged for every subject. Then, the acquisitions for all subjects were averaged to highlight the differences between regions [Figs. 8(b) and 8(c)] and the interindividual variability based on the standard deviation (blue bands).

### 3.5 Oximetry Measurements

Ocular oximetry assessments, reported as ${\mathrm{StO}}_{2}$, were made using the spectra from the different locations in the eye fundus of the eight healthy subjects. This was performed as a demonstration of a concrete application stemming from targeted spectral analysis and not as a validation of ocular oximetry per se. ${\mathrm{StO}}_{2}$ was calculated based on previously described methods^26^^,^^32^ for each acquired spectrum and averaged over the acquisition at a specific location for each subject. The difference in average ${\mathrm{StO}}_{2}$ in the parafovea (Fig. 8, location A) and the optic nerve head (Fig. 8 location B) was compared for each subject (Table 1). Results showed a greater interindividual variability in ${\mathrm{StO}}_{2}$ in the parafovea (ranging from 30.4% to 58.4%) than in the optic nerve head (ranging from 62.1% to 69.7%). A Wilcoxon signed-rank test was performed to establish the statistical significance of the difference between the measurements made at the two locations (p=0.004).

**Table 1:** ${\mathrm{StO}}_{2}$ values at two locations in the eye fundus of healthy subjects, showing a significant difference between values in the parafovea and the optic nerve head (Wilcoxon signed-rank test, p-value=0.004).

Subject ID	Blood oxygen saturation ${\mathrm{StO}}_{2}$ (%)		
	Parafovea (A)	Optic nerve head (B)	Difference (B − A)
1	31.4	69.3	37.9
2	32.1	62.3	30.2
3	58.0	63.5	5.5
4	39.3	68.4	29.1
5	52.7	69.7	17.0
6	58.4	67.6	9.2
7	48.2	68.8	20.6
8	30.4	62.1	31.7

An example of the stability and reproducibility of the ${\mathrm{StO}}_{2}$ measurements is shown by comparing 13 measurements taken over a 5-s acquisition in the optic nerve head of a patient (Fig. S4 in the Supplementary Material). The mean ${\mathrm{StO}}_{2}$ was 70.24%, with a standard deviation of 1.28%.

## 4 Discussion

This work presents a multimodal system with the ability to concurrently and continuously perform imaging and targeted spectroscopy in the eye fundus. Spectral analysis can be used in the context of reflectance and fluorescence and has the potential to be applied for a broad range of applications and biomarkers, including ocular oximetry.

The eye fundus is generally regarded as a highly heterogeneous structure, with respect to both features (large blood vessels, microvasculature, macula, fovea, optic nerve head, etc.) and compositions (components, biomarkers, etc.). Biomarkers can be broadly dispersed throughout the tissue (e.g., β-amyloid plaques in the retina of Alzheimer’s patients)^33^ or localized in specific regions (e.g., cotton wool spots and hemorrhages for patients with DR^34^). With targeted spectroscopy, a pointing LED is used to identify the region for which spectral analysis is to be performed, enabling sensitive spectral analysis of specific features of the eye fundus. Thus, it was important to demonstrate that the projected area of the LED on a surface or on the eye fundus corresponded to the actual ROSA and that the spectra acquired were distinct from region to region, which was done through the use of a reference target (Figs. 4 and 5) and of a model eye (Figs. 6 and 7). These *in vitro* demonstrations of the capacity of the system to provide valuable spectral information from diffuse reflectance and fluorescence at specific targeted locations highlight important features of the technology.

The detection of biomarkers in the retina from absorbance or fluorescence spectra is dependent on many factors, the most important being the capacity to isolate specific spectral signatures and to detect weak signals. Thus, there is a need for high sensitivity, high spectral resolution, and short acquisition speed, all characteristics of targeted spectroscopy. Wide-range and high-resolution spectra from the ROSA and images of the eye fundus can be acquired within tens of milliseconds. For example, for the 5-s acquisitions presented in Fig. 8, spectra were recorded every 25 ms for the optic nerve head and every 175 ms for the parafovea (LED intensity of 1 mW). A few points are important to note. Firstly, the variation in integration times and, by extent, in the speed of a single-spectrum acquisition between the two regions was set to ensure that enough signal was recovered for high-sensitivity spectral analysis. Secondly, analysis of a small region of the eye fundus (rather than of the whole field of view) reduces the number of photons being analyzed. Hence, a longer integration time and/or a stronger illumination source is required to obtain enough signal for measurements in many features of the eye fundus. Thirdly, using a high-intensity light source (e.g., xenon light^17^^,^^18^ or brighter LED) can lead to shorter integration times in fundus spectroscopy; however, this also shortens the acceptable exposure time (based on eye safety and subject comfort) and does not enable continuous, time-resolved acquisitions.

Other systems, such as hyperspectral imaging^35^ and vis-OCT,^25^ face the same compromise between spectral resolution and acquisition speed. Desjardins et al.^22^ reported hyperspectral imaging in under 3 s for a 30-deg field of view and a wavelength range of 500 to 600 nm with 2 and 5 nm steps, and later in under 1 s for a range of 450 to 900 nm in steps of 5 nm.^36^ In hyperspectral systems, a compromise stems from the inherent delay associated with changing the spectral window of the filter. Systems that use hyperspectral snapshot cameras must choose between spatial resolution and spectral resolution. For example, Lemmens et al.^37^ acquired a spectrum from 460 to 620 nm, 10-nm bandwidth, and 272×512  pixels images from a single snapshot. Different groups have demonstrated the ability of vis-OCT to extract metabolic information from visible light spectra. Pi et al.^32^ measured retinal blood oxygen saturation *in vitro* and in rats with an acquisition time of 17 s at 50 kHz A-line speed for a 510 to 610 nm range (laser power was not reported). Recently, Song et al. showed that the reflectance signal from vis-OCT, with an acquisition time of 2.6 s at 50 kHz A-line speed and a laser power at the pupil of 0.25 mW for a 545 to 580 nm range, could better distinguish glaucoma subjects from normal eyes than thickness measurements from OCT. In another paper, Song et al.^38^ reported vis-OCT acquisitions of <6  s for a field of view of 3  mm×7.8  mm and a wavelength range of 545 to 580 nm. Long acquisition times require the patient to have a stable fixation while being subjected to high irradiance for the whole duration, which is a major drawback and represents a challenge for the clinical adoption of these types of technologies. In addition, both technologies have lower sensitivity to local spectral variation than targeted spectroscopy because their spectral signal is coming from the whole field of view.

The capacity to identify a broad range of biomarkers (be they associated with the presence of a compound or a change in structural organization of the tissue) from their spectral signatures opens the door to a wide range of applications. One such example is ocular oximetry, the assessment of blood oxygen saturation in the different tissues of the eye fundus. There is accumulating evidence linking oxygen saturation in specific regions of the eye fundus to a number of diseases, including glaucoma,^39^^–^^45^ DR,^39^^,^^40^^,^^46^^–^^49^ AMD,^50^ vascular occlusions,^39^^,^^40^^,^^51^^,^^52^ and multiple sclerosis.^53^ Thus, it is an increasingly important biomarker.

The evaluation of blood oxygen saturation in the eye has been attempted through methods such as dual-wavelength analysis^54^ and hyperspectral imaging.^55^ In their current state, the limited sensitivity of these approaches only allows for relative assessment of oximetry and is mostly limited to large blood vessels of the eye fundus. Over the years, new methods were developed using broader spectral bandwidths, showing improvements in sensitivity and accuracy and enabling measurements in the retinal tissues.^19^^,^^20^^,^^26^^,^^56^ In this study, we used an ocular oximetry algorithm that has previously been validated using *in silico* and *in vitro* experiments^26^ as an example of how algorithms based on the spectral analysis of biomarkers could be combined with targeted ocular spectroscopy.

The results presented in Fig. 8 demonstrate how targeted spectroscopy can provide spectral profiles specific to different features of the eye fundus, in this case, the optic nerve head and the parafovea. The evaluation of blood oxygen saturation also showed that measurements made in these different regions led to different values of ${\mathrm{StO}}_{2}$ (Table 1), with lower oxygen saturation and greater interindividual variability observed in the parafovea. This can be explained by a number of factors. Firstly, the fovea is typically the region in the retina with the highest density of photoreceptor cells and with the broadest range of melanin between individuals, leading to higher absorbance and variability compared with the optic nerve head.^57^ Secondly, a significant portion of the oxygen supplied to the foveal region comes from the choroid through the diffusion of molecular oxygen, which is not detected as oxyhemoglobin. Thirdly, OCT-angiography studies have shown that vascular density decreases as we approach the fovea,^58^ leading to lower blood volumes and greater relative oxygen utilization. This likely explains the decreasing intensity of the hemoglobin spectral components and the lower oxygen saturation seen in acquisitions made in this region. Spectra from the optic nerve head displayed a stronger contribution from hemoglobin (and specifically oxyhemoglobin); the nerve fibers, which are the main constituents of the optic nerve head,^59^ act as diffusers for the main local absorber, which is hemoglobin. Interestingly, some ${\mathrm{StO}}_{2}$ measurements made in the parafovea were lower than blood oxygen saturation values from large retinal veins reported in the literature.^41^^,^^49^ Although the present work does not aim to perform a validation of the oximetry algorithm used, some factors could explain this difference. The oxygen saturation of the blood in large retinal veins corresponds to the weighted average of oxygen saturation of blood returning from various regions of the eye fundus (some with higher ${\mathrm{StO}}_{2}$, some with lower ${\mathrm{StO}}_{2}$). Thus considering the heterogeneity in the retinal tissue, one expects the local blood oxygen saturation in the retinal tissue (such as in the parafovea) to deviate from the mean value (the extent of this deviation is yet to be determined).

On the other hand, the *in vitro* fluorescence results presented open the door to another set of potential applications. Different fluorophores are naturally present in the eye fundus, and they can contribute to diagnosing retinal pathologies. For example, lipofuscin accumulation is associated with AMD and macular dystrophies such as Best and Stargardt disease.^60^ Typically, the fundus autofluorescence capabilities of a camera enable the estimation of the amount of molecules present. Our technique enables the user to target specific regions of interest previously identified by wide-field fluorescence and then acquire a full emission spectral profile of the molecules present. This information could be of use for diagnostic purposes. For example, Feldman et al. reported that the emission profile of A2E, a component of the RPE, can provide insightful information on the presence of AMD.^12^

## 5 Conclusion

The system presented here enables the concurrent and continuous acquisition of images and diffuse reflectance or fluorescence spectra from targeted regions of the eye fundus. The targeted nature of the method was tested in vitro using reference targets and a model eye. Additional testing was performed *in vivo* on the parafovea and the optic nerve head of healthy subjects. In all cases, distinct spectral profiles were acquired in the different regions tested. Finally, an ocular oximetry algorithm was implemented to spectra acquired *in vivo*, showing an example of a potential application for the technology. Targeted ocular spectroscopy has the potential to assess the presence of different chromophores and fluorophores, such as hemoglobin, oxyhemoglobin, melanin, and lipofuscin, associated with disease progression. This could open the door to change the way we diagnose and treat eye diseases, and targeted ocular spectroscopy could become an increasingly important tool in eye care in the coming years.

## Data Availability

Data underlying the results presented in this paper are not publicly available at this time but may be obtained from the authors upon reasonable request.
