# Supplementary material for: Targeted spectroscopy in the eye fundus
Source: J Biomed Opt. 2023 Dec 15;28(12):126004. doi: 10.1117/1.JBO.28.12.126004 (PMC10725981; doi:10.1117/1.JBO.28.12.126004)
Supplement: Supplementary file 1 [file JBO_028_126004_SD001.pdf]

Targeted spectroscopy in the eye fundus

Nicolas Lapointe,[a] Cléophace Akitegetse,[a] Jasmine Poirier,[a] Maxime Picard,[a] Patrick Sauvageau,[a] Dominic Sauvageau[a,b,*]

[a]Zilia Inc, Québec, QC, Canada
[b]University of Alberta, Chemical and Materials Engineering, Edmonton, AB, Canada

SUPPLEMENTARY MATERIAL

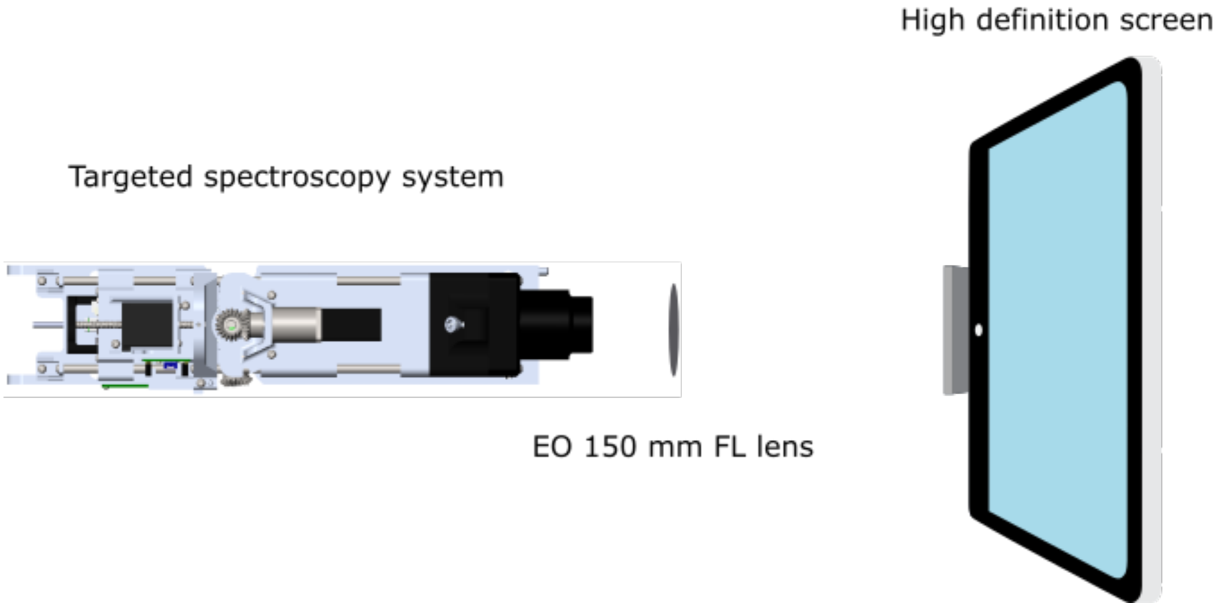

Figure S1. Configuration for the validation of the region of spectral acquisition. The targeted spectroscopy system is placed in front of a high-definition screen. A lens is placed in between to relay the image onto the sensor. The screen is used to display different and customizable targets to validate the system.

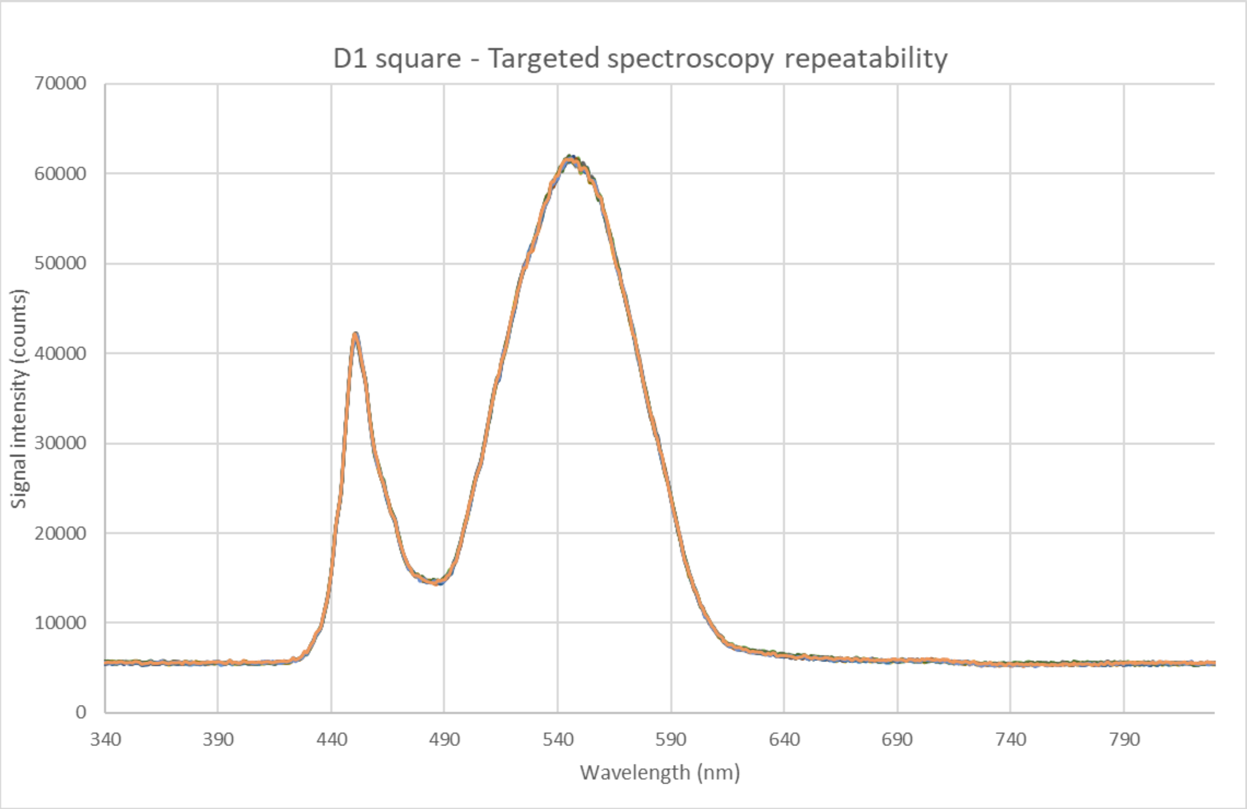

Figure S2. Reproducibility of spectral acquisitions in region D1 shown in Figure 4a. Data shown represents the overlay of 8 raw spectra acquired from region D1 of Figure 4a.

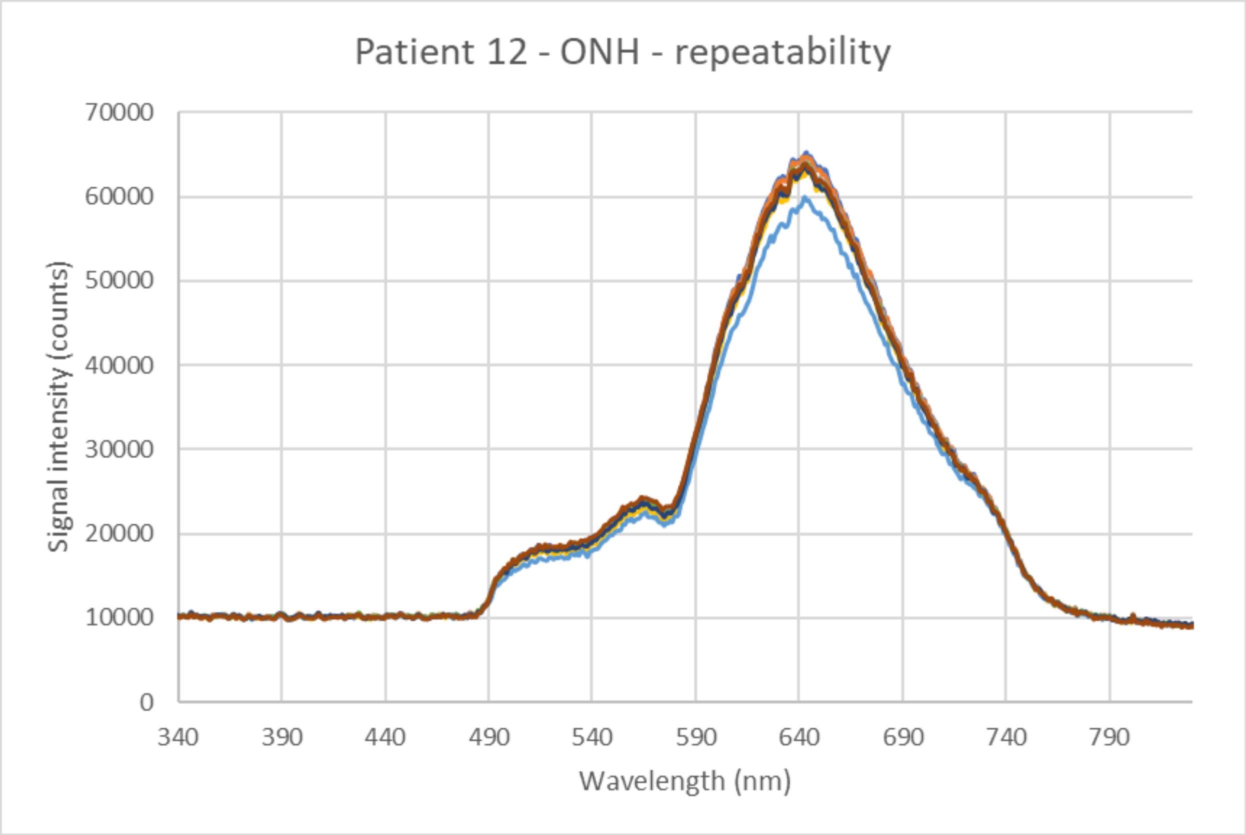

Figure S3. Reproducibility of spectral acquisitions in the optic nerve head. Data shown represents the overlay of 13 raw spectra acquired from a 5-sec acquisition. These spectra were used to obtain the absorbance of a given targeted region of the optic nerve head and to calculate StO_2.

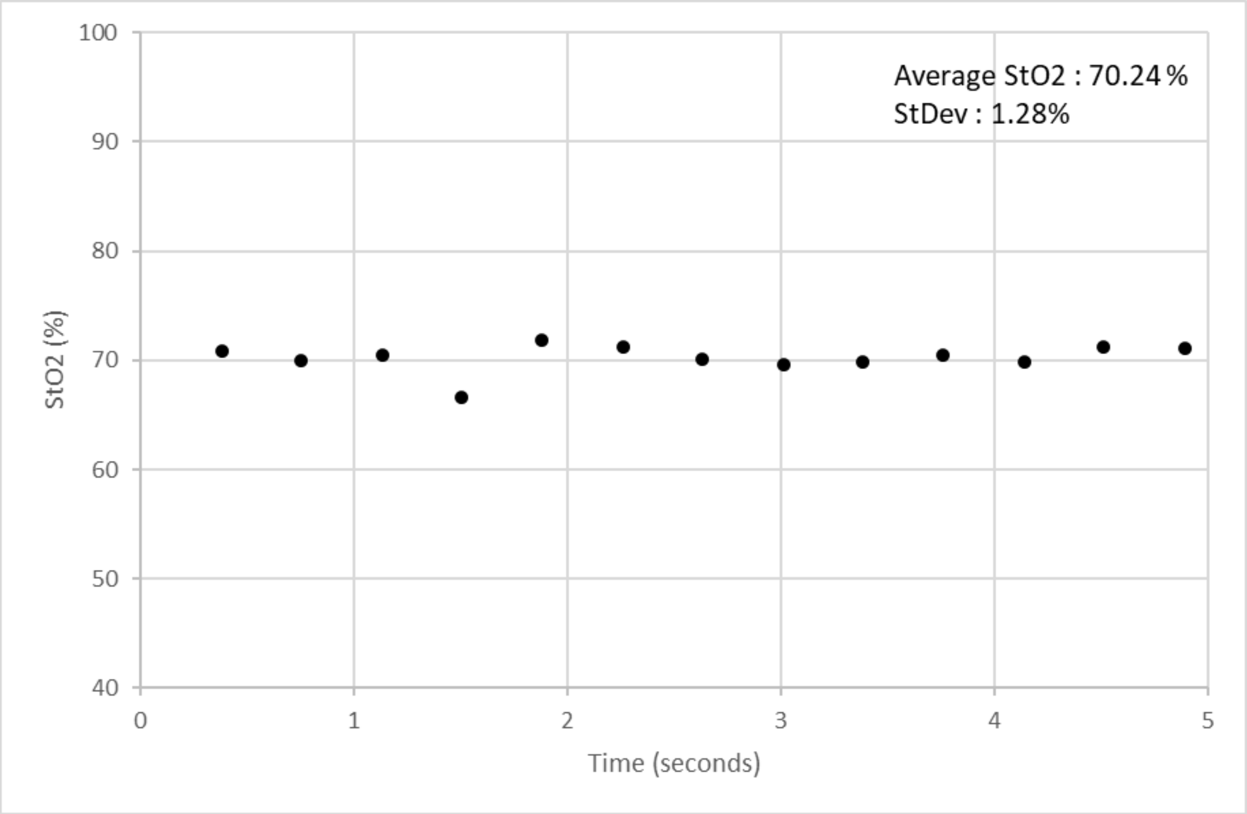

Figure S4. Ocular oximetry acquisition in the optic nerve head. 5- second acquisition from the optic nerve head totalling 13 reported measurements. Mean StO_2 was 70.24% with a standard deviation of 1.28%.
